# Divergent biophysical controls of aquatic CO_2_ and CH_4_ in the World’s two largest rivers

**DOI:** 10.1038/srep15614

**Published:** 2015-10-23

**Authors:** Alberto V. Borges, Gwenaël Abril, François Darchambeau, Cristian R. Teodoru, Jonathan Deborde, Luciana O. Vidal, Thibault Lambert, Steven Bouillon

**Affiliations:** 1 Université de Liège, Unité d’Océanographie Chimique, Institut de Physique (B5), B-4000, Belgium; 2 Laboratoire Environnements et Paléoenvironnements Océaniques et Continentaux (EPOC), CNRS, Université Bordeaux 1, Avenue des Facultés, 33405 Talence, France; 3 Programa de Geoquímica, Universidade Federal Fluminense, Outeiro São João Batista s/n, 24020015, Niterói, RJ, Brazil; 4 Katholieke Universiteit Leuven, Department of Earth and Environmental Sciences, Celestijnenlaan 200E, B-3001 Leuven, Belgium; 5 Laboratório de Ciências Ambientais, Centro de Biociências e Biotecnologia, Universidade Estadual do Norte Fluminense – UENF, Av. Alberto Lâmego, Parque Califórnia, CEP 28013602, Campos dos Goytacazes, RJ, Brazil

## Abstract

Carbon emissions to the atmosphere from inland waters are globally significant and mainly occur at tropical latitudes. However, processes controlling the intensity of CO_2_ and CH_4_ emissions from tropical inland waters remain poorly understood. Here, we report a data-set of concurrent measurements of the partial pressure of CO_2_ (pCO_2_) and dissolved CH_4_ concentrations in the Amazon (n = 136) and the Congo (n = 280) Rivers. The pCO_2_ values in the Amazon mainstem were significantly higher than in the Congo, contrasting with CH_4_ concentrations that were higher in the Congo than in the Amazon. Large-scale patterns in pCO_2_ across different lowland tropical basins can be apprehended with a relatively simple statistical model related to the extent of wetlands within the basin, showing that, in addition to non-flooded vegetation, wetlands also contribute to CO_2_ in river channels. On the other hand, dynamics of dissolved CH_4_ in river channels are less straightforward to predict, and are related to the way hydrology modulates the connectivity between wetlands and river channels.

There is an increasing recognition of the importance of inland waters (streams, rivers, lakes and reservoirs) in global budgets of CO_2_ and CH_4_. According to the most recent estimate, the CO_2_ emission from inland waters totals 2.1 PgC yr^−1^^1^ which is equivalent to the ocean or land CO_2_ sinks^2^. The global emission of CH_4_ to the atmosphere from freshwater ecosystems of 103 TgCH_4_ yr^−1^^3^ is significant when compared to all other natural (220–350 TgCH_4_ yr^−1^) and anthropogenic (330–335 TgCH_4_ yr^−1^) CH_4_ emissions^4^. Wetlands are among the largest natural CH_4_ sources to the atmosphere ranging between 175 and 220 TgCH_4_ yr^−1^^4^, although pristine freshwater wetlands sequester carbon (C) below ground as organic matter at a rate of ~0.8 PgC yr^−1^^5^. We adopt, here, the common definition of wetlands as habitats with continuous, seasonal, or periodic standing water or saturated soils^6^. The total estimated CO_2_ emission from rivers and streams of 1.8 PgC yr^−1^^1^ is mostly related to tropical areas that account for 1.4 PgC yr^−1^ (78%). However, the CO_2_ data distribution is skewed towards temperate and boreal systems in the Northern Hemisphere, and data in several tropical basins (including the Congo) were derived from interpolation from adjacent basins rather than actual measurements. About 49% of the CH_4_ emission to the atmosphere from freshwater ecosystems occurs in the tropics, although, there is equally a strong under-representation of tropical inland waters in global estimates, whereby the most recent global synthesis resorted to extrapolating CH_4_ fluxes from temperate rivers^3^.

The C emissions from inland waters result from complex interactions between hydrology, biogeochemical processing within the aquatic environment and connectivity with riparian zones and the watershed. The CO_2_ emissions from inland waters have been traditionally interpreted as mainly resulting from the *in-situ* degradation of organic C from non-flooded land (that is, *terra firme*)^7^,^8^,^9^,^10^,^11^,^12^,^13^,^14^,^15^. Yet, other sources of CO_2_ could also contribute to CO_2_ emissions from inland waters. In lakes, there is an increasing recognition of the role of hydrological inputs of CO_2_ (rivers and groundwaters) in sustaining CO_2_ emissions to the atmosphere^16^,^17^,^18^,^19^,^20^. In rivers, the contribution of groundwater inputs of CO_2_ to riverine CO_2_ emissions is also recognized as particularly important in headwaters^21^,^22^. There is also an increasing recognition of the inputs of C from wetlands in sustaining CO_2_ and CH_4_ emissions to the atmosphere from rivers and lakes. Wetlands contribute to CO_2_ emissions through the respiration from flooded roots of vegetation and by providing labile organic C to sustain bacterial degradation^23^,^24^. In the Central Amazon basin, CO_2_ and CH_4_ emissions from floodplain lakes^23^,^25^ and from river channels^24^,^26^ have been attributed to C from wetlands (flooded forest and macrophytes) in addition to non-flooded terrestrial organic C. This was established with a mass balance approach of organic C^23^,^26^, high-resolution pCO_2_ distributions^24^, and stable-isotope signatures of organic C. In African rivers, spatial patterns of pCO_2_ and CH_4_ relate to the distribution of the fraction of wetland in the catchment within a given system (Congo and Zambezi) and across different basins^27^,^28^. However, both non-flooded terrestrial biomass and wetlands contribute to CO_2_ emissions from inland waters and their relative importance remains uncertain and has not yet been quantitatively resolved^27^,^29^. This is in part due to the absence of specific molecular tracers for terrestrial organic matter, since numerous plants are common in flooded and non-flooded forests^30^. On the other hand, stable isotopes allow to trace organic matter from floating macrophytes that frequently have a C_4_ signature^31^, while non-flooded C_4_ grasslands have been found to contribute little to organic matter transported by rivers even in catchments where they occupy extensive areas^32^. The relative contribution of flooded and non-flooded biomes to riverine CO_2_ emissions will vary from one basin to another as a function of climate^27^. It will also vary within a given basin with a dominance of non-flooded terrestrial inputs in headwaters and highlands and an increased contribution of wetlands in lowlands^24^,^27^,^29^,^31^. In the Amazon basin, wetlands have been conclusively shown to be hotspots of CH_4_ emission compared to river channels^25^,^33^,^34^.

Here, we compare the CO_2_ and CH_4_ distributions in lowland river channels of the two largest rivers in the World and in the tropics, the Amazon and the Congo (Table 1), using a data-set of concurrent pCO_2_ and CH_4_ concentration measurements in river channels (Fig. 1, Table 2). We acknowledge that there are several other data-sets of pCO_2_ and CH_4_ in Amazonian aquatic systems^29^ but we focus on direct measurements of pCO_2_ (not calculated from pH and TA that are highly biased in acid waters^35^) concurrent with dissolved CH_4_ measurements (most other studies are based on either one dissolved gas or the other, but not both). The aim of this study is to determine the extent to which the patterns of CO_2_ and CH_4_ differ or converge in these two tropical giant water bodies.

## Results

The pCO_2_ values spanned two orders of magnitude in the Amazon (70 to 16,880 ppm) and one order of magnitude in the Congo (1090 to 22,900 ppm) (Fig. 2a). The CH_4_ concentrations spanned four orders of magnitude in the Amazon (11 to 189,100 nmol L^−1^) and three orders of magnitude in the Congo (22 to 71,430 nmol L^−1^) (Fig. 2b). Data were aggregated into mainstem (MS), large and small tributaries (T > 100 m and T < 100 m width, respectively^11^,^36^). The pCO_2_ values significantly increased from the mainstem to the small tributaries in the Amazon (Kruskal-Wallis (KW) test, *p* = 0.0001) and in the Congo (KW test, *p* < 0.0001). The same pattern was observed for CH_4_ concentrations in the Amazon (KW test, *p* < 0.0001) and in the Congo (KW test, *p* < 0.0001). In the mainstem, large and small tributaries of both Amazon and Congo, the median pCO_2_ and CH_4_ (Fig. 2a,b) were distinctly above atmospheric equilibrium of ~390 ppm and ~2 nmol L^−1^, respectively. The pCO_2_ in the Amazon mainstem was significantly higher than in the Congo mainstem, but pCO_2_ values were not significantly different in large and small tributaries (Fig. 2a). The CH_4_ in the mainstem, large and small tributaries were significantly higher in the Congo than in the Amazon (Fig. 2b). The median CH_4_ in the Congo was three to four times higher than in the Amazon, for mainstem/small tributaries and large tributaries, respectively. For a given pCO_2_ value, CH_4_ concentrations were systematically higher in the Congo than in the Amazon (Fig. 3a–c).

## Discussion

### The contribution of wetlands to CO_2_ emissions in the Amazon, Congo and across tropical rivers

The pattern of higher pCO_2_ values in streams compared to rivers in the Amazon and the Congo (Fig. 2) is consistent with an analysis of global averages^36^ and also with the regional studies in part of the Congolese “Cuvette Centrale”^37^ and in the Oubangui sub-catchment^38^. Higher CH_4_ and CO_2_ concentrations in tributaries than in the mainstem were also reported in the Paraguay River^39^. The higher pCO_2_ in the mainstem of the Amazon than in the Congo in their lowland regions could be due to the higher wetland coverage (Table 1), since organic and inorganic C from wetlands has been shown to partly sustain the CO_2_ emission from the Central Amazon mainstem and floodplains^24^,^26^. In order to expand the range of wetland coverage, we included pCO_2_ data acquired in four other African rivers^27^ (Fig. 4). In the small and large tributaries and mainstem, pCO_2_ was positively correlated to wetland coverage across these six tropical rivers, confirming the contribution of wetland C in partly sustaining CO_2_ emissions from lowland tropical river channels^24^,^26^,^27^. These positive correlations between pCO_2_ and wetland coverage do not necessarily imply that wetlands are the sole drivers of CO_2_ in river channels. As previously noted, semi-arid rivers such as the Tana that are virtually devoid of wetlands are CO_2_ sources to the atmosphere, although less intense than other tropical rivers, implying that non-flooded land also sustains CO_2_ emissions from river channels^27^. The relative importance of non-flooded land and wetlands in sustaining riverine CO_2_ emissions remains uncertain and has not yet been quantitatively resolved^29^.

### Several hypotheses can explain the different behavior of CH_4_ in the Amazon and Congo river channels

Although in African rivers average CH_4_ concentrations correlate with wetland coverage^27^, CH_4_ concentrations were significantly higher in the Congo than in the Amazon river channels (Fig. 2), despite the fact that the Amazon has a higher wetland coverage (Table 1). Further, the correlations of CH_4_ and pCO_2_ are different in the Amazon and Congo river channels (Fig 3). In small streams (T < 100 m), the strong positive relationship between CH_4_ and pCO_2_ in both rivers indicates a common origin. It might indicate a stronger contribution of CO_2_ production from anaerobic organic matter degradation compared to aerobic respiration, and that both CO_2_ and CH_4_ production are related to C processing within wetlands. Small streams receive higher contributions from groundwater that are rich in CO_2_^21^,^22^. However, data in African rivers show that groundwater had an extremely low CH_4_ content^27^,^40^. While groundwater input certainly contributes to high CO_2_ in small streams it cannot explain the extremely high CH_4_ in small streams. Consequently, the strong correlation between pCO_2_ and CH_4_ in small streams (Fig. 3b) indicates that groundwater inputs are probably not the major drivers of the high pCO_2_ values at our sampling sites in lowland regions. In the mainstem, CH_4_ is only weakly positively correlated to pCO_2_ in the Congo, while a weak negative relation is observed in the Amazon. This might indicate that in the well mixed and well oxygenated Amazon mainstem, there is a stronger contribution to CO_2_ production of aerobic respiration fueled by both non-flooded and wetland organic matter^41^, while CH_4_ is lost by emission to the atmosphere and bacterial oxidation. In large tributaries (T > 100 m) an intermediary situation is observed in the Amazon, while in the Congo, CH_4_ and pCO_2_ remain strongly correlated. These fundamental differences in the dynamics of CH_4_ in these two rivers can be further examined by invoking several hypotheses.

**First**, the Congo flooded wetland is in majority flooded forest^42^ and there are no temporary floodplain lakes but only a handful of relatively large permanent lakes (Mai-Ndombe (2,300 km^2^), Tumba (765 km^2^)). In the Central Amazon, on the other hand, flooded forest accounts for 80% of the maximum flooded wetland extent, and the remaining 20% corresponds to temporary and permanent lakes (7% of open water and 13% of floating macrophytes). There are 6,500 floodplain lakes from 52.5°W to 70.5°W along the floodplain fringing the Amazon mainstem plus 2,300 lakes on the major tributaries, totaling a surface area of 10,400 km^2^^43^. Floodplain lakes are abundant downstream of the confluence of the Negro and Solimões Rivers, while upstream wetland is dominated by flooded forest. Floodplain lakes are characterized by high gas transfer velocity (*k*) values^44^,^45^, that promote the evasion of CH_4_ to the atmosphere and water oxygenation that will favor bacterial CH_4_ oxidation. In the Congolese and Amazonian flooded forest, *k* values should be low due to wind shielding and moderate diurnal water and air temperature variations below the dense canopy, and the release by the flooded plants of hydrophobic organic matter, which might behave as surfactants. This limits CH_4_ loss by evasion to the atmosphere and by bacterial oxidation (low oxygen levels).

**Second**, local upland runoff is the main source of the wetland water in the Congo, and not flooding by riverine overflow as in the Amazon^46^. This unidirectional flow pattern will promote the transport of the CH_4_ produced in the flooded forest towards the small and large river channels of the Congo, unlike in the Central Amazon where during rising water and high water, the water transport is from the river channels towards the wetlands. It is during rising water and high water that floating macrophytes grow and their biomass peaks^47^. This corresponds to the period of highest CH_4_ emissions^33^, and presumably also highest CH_4_ production, when the water transfer from wetlands to the river channels is blocked by flooding. The same applies to flooded forest where CH_4_ emissions were also found to be highest during high water^33^.

**Third**, the Congo wetlands are mostly permanently flooded unlike the Amazon floodplains that are seasonally flooded. Permanently flooded wetlands are known to be stronger CH_4_ emitters and presumably CH_4_ producers than seasonal flooded wetlands^48^,^49^.

**Fourth**, in the Congo, floating macrophytes (mainly *Vossia cuspidata*) commonly occur along channel edges and within channels, and form large meadows in streams, rivers and mainstem, in all types of waters (white and black). Floating macrophytes are known to host high CH_4_ production and emission^25^,^33^,^34^ that will be directly delivered into the Congo river channels. This does not occur in the Amazon where macrophytes are mainly present in floodplain lakes and do not occur in large tributaries and the mainstem due to important depth and strong currents. This is consistent with the higher CH_4_ concentrations in the Congo than in the Amazon mainstem for pCO_2_ values >7000 ppm (Fig. 3a). The CH_4_ released by floating macrophytes in the Amazonian wetland lakes will be lost locally by evasion to the atmosphere and CH_4_ oxidation (see above), and little dissolved CH_4_ will be transported to the river channels.

All these differences are related to the smaller water height variations in the Congo mainstem (3–4 m) compared to the Amazon (10–12 m). The Congo basin straddles on the equator, and the dry season on the Northern part of the basin is compensated by the rainy season on the Southern part of the basin, and *vice-versa*, leading to a regulation of seasonal water height variations^50^. These different hypotheses need to be tested and verified although this would require a detailed investigation of the hydrology and wetland habitat mapping that are lacking in the Congo where research on aquatic biogeochemistry and ecology was largely abandoned since the early 1960’s compared to the Amazon that has been the subject of continued investigations for more than five decades.

### Re-evaluation of CO_2_ emissions from tropical rivers and streams

The total CO_2_ emission from river and streams estimated by Raymond *et al*.^1^ of 1.8 PgC yr^−1^ is mostly related to tropical areas that account for 1.4 PgC yr^−1^ (78%). However, the data coverage in the tropics was lower than for temperate and boreal regions, and data in several basins (including the Congo) were derived from interpolation from adjacent basins rather actual measurements. Furthermore, only one value of pCO_2_ was used for the whole watershed while pCO_2_ values increase in lower order streams as shown here (Fig. 2) and across the United States^15^. For African rivers we have previously shown that the Raymond *et al*.^1^ dataset underestimated CO_2_ fluxes in five basins where new direct pCO_2_ measurements were recently made^27^. Although based on a limited number of river basins, we used the regressions in Fig. 4 as a first attempt to re-evaluate CO_2_ emissions from tropical rivers and streams globally. The river basins shown in Fig. 4 cover a large range of size, climate, and land and wetland cover typical of those encountered in tropical areas. The resulting flux for the tropics is 1.8 ± 0.4 PgC yr^−1^, *i.e*. 25% higher than the value originally computed by Raymond *et al*.^1^. While additional data will be required to further refine global estimates, this exercise confirms the importance of CO_2_ emissions from rivers in tropical areas.

In conclusion, the analysis of data in river channels in six tropical rivers including the two largest ones (Amazon and Congo) reported here demonstrates that large-scale patterns in pCO_2_ across different basins can be apprehended with a relatively simple statistical model related to the extent of wetlands within the basin. Dynamics of dissolved CH_4_ in river channels are less straightforward to predict, and appear to be related to the way hydrology modulates the connectivity between wetlands and river channels. The differences we have highlighted in CH_4_ concentration in the river channels of the Amazon and Congo should translate into same differences in CH_4_ emissions, since in river channels the diffusive CH_4_ emission is much higher than CH_4_ ebullition flux in both rivers^27^,^51^. This is not the case in wetlands where ebullition represents the majority of the CH_4_ emission to the atmosphere^25^,^52^. In the Amazon basin, overall aquatic CH_4_ emissions are dominated by wetlands^25^, while equivalent estimates are unavailable for the Congo basin.

## Methods

### Study site characteristics

**Figure 1:**
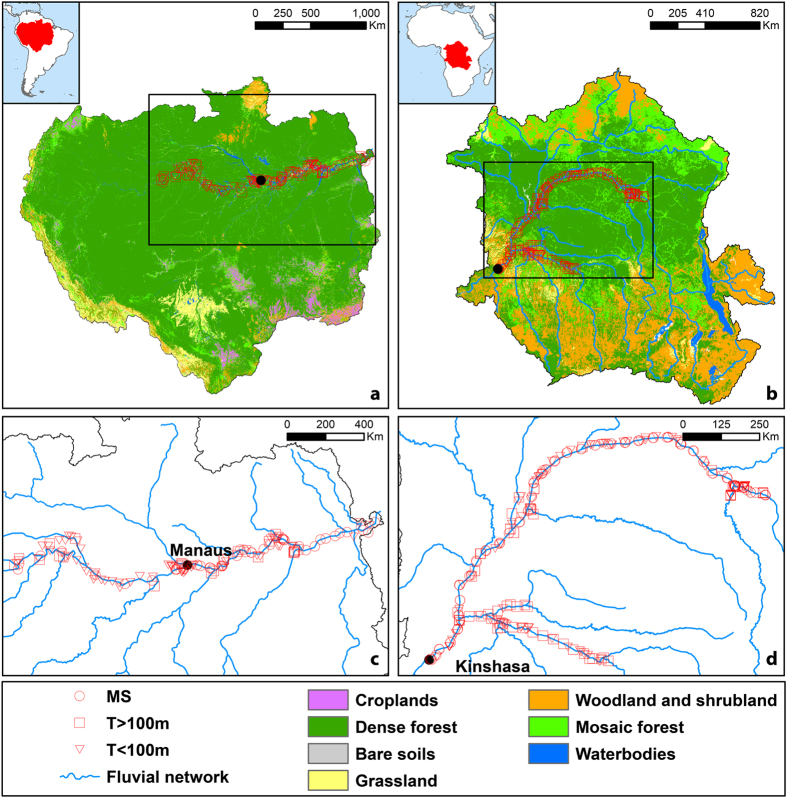
Location of sampling stations in the Amazon and Congo at the scale of the whole basin overlain on the land cover (a,b), and a zoom overlain on the main rivers (d,e). Maps were generated with ArcGIS using publically available spatial datasets^60^,^61^. MS = mainstem. T > 100m = large tributaries. T < 100m = small tributaries.

**Figure 2:**
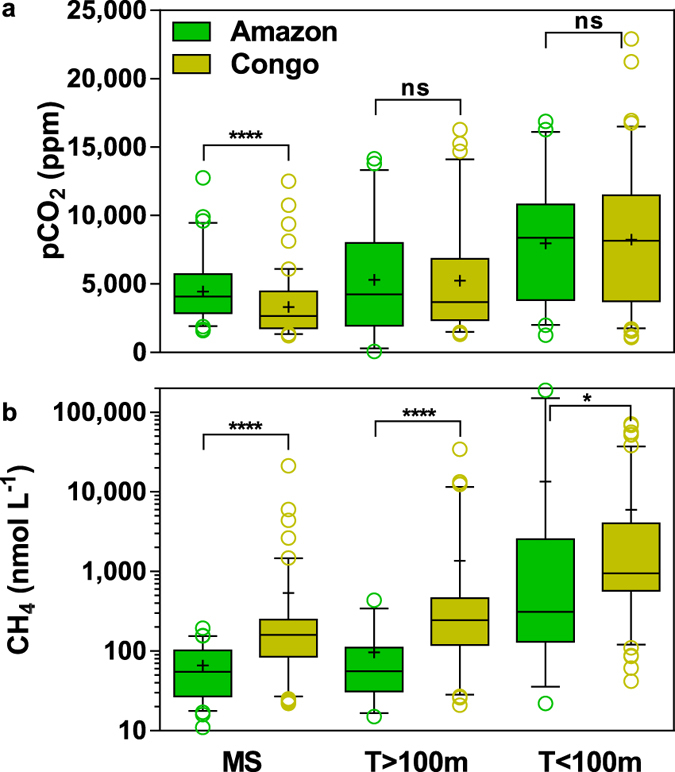
Box and whisker plots of pCO_2_ (a) and CH_4_ (b) in the Amazon and Congo. The box spans the interquartile range (25–75 percentiles), whiskers correspond to 5–95 percentiles, horizontal bar to median, cross to average, and circles to outliers. Differences were tested with a Mann Whitney test at 0.05 confidence interval level, where **** corresponds to *p* < 0.0001, * to *p* = 0.0278, and ns to not significant. MS = mainstem. T > 100m = large tributaries. T < 100m = small tributaries.

**Figure 3:**
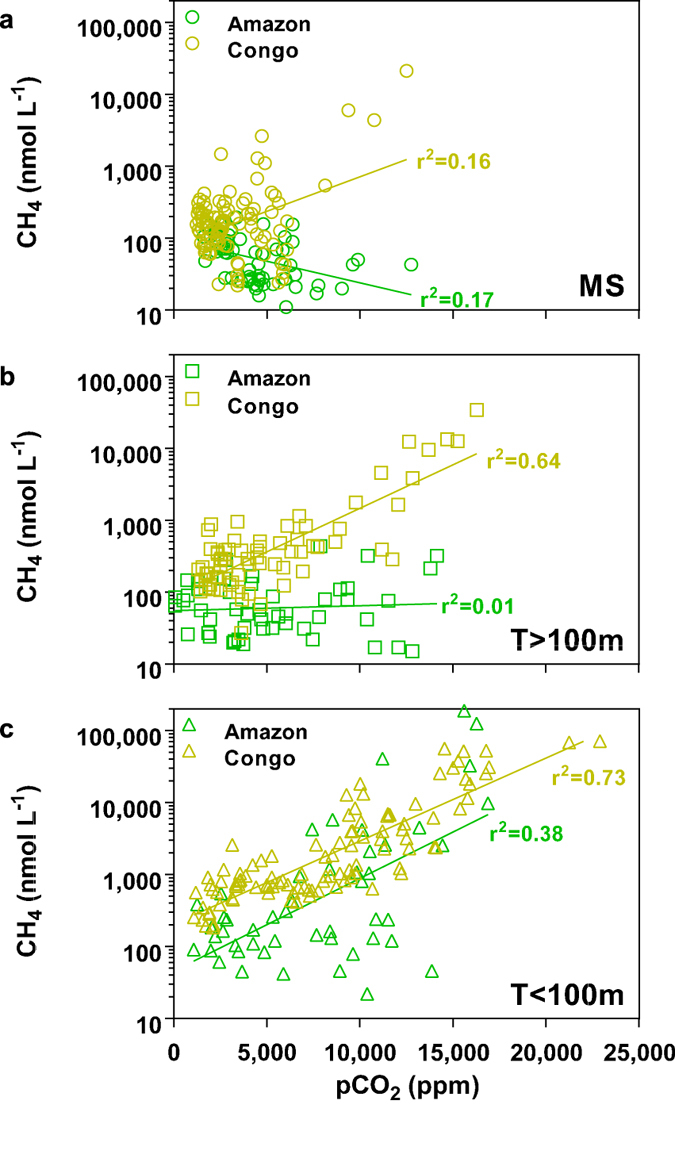
Log of CH_4_ concentration as function of pCO_2_ in the mainstem (MS) (a), the large tributaries (T > 100 m) (b), and small tributaries (T < 100 m) (c) of the Amazon and Congo basins. Lines correspond to the linear regressions of log transformed CH_4_ as a function of pCO_2_.

The Amazon and Congo are the first and second largest rivers in the World, respectively, in terms of catchment area and freshwater discharge (Table 1). The Amazon basin is on average ~1 °C warmer and has an annual precipitation about two times higher than in the Congo. This leads to a specific discharge that is also much higher in the Amazon than in the Congo. The higher precipitation can also explain the higher coverage of the basin by evergreen forest (dense and mosaic) in the Amazon (87%) than in the Congo (67%), where conversely savannah (shrubland and grassland) is more abundant (30%), in particular in the northern and southern rims of the catchment (Fig. 1). Consequently, average above ground biomass is higher in the Amazon than in the Congo. The Amazon and Congo basins include the largest tropical wetlands in the World, with annual mean flooded area of 730,000 and 360,000 km^2^, respectively^25^,^42^.

### Field data collection

Data were acquired during 5 cruises in the Amazon and 6 cruises in the Congo covering different stages of the annual flood cycle (Table 2). The pCO_2_ in the Amazon was measured with an equilibrator^53^ coupled to an infra-red gas analyzer (IRGA), as described in detail by Abril *et al*.^24^. The pCO_2_ in the Congo was measured with both an equilibrator (in the mainstem and largest tributaries) and with a syringe headspace technique (in the mainstem and large and small tributaries) with an IRGA, as described in detail by Borges *et al*.^27^. Both approaches were inter-calibrated and compared very well^35^. Only the data acquired with a syringe headspace technique in the Congo are presented here. Samples for the determination of CH_4_, were conditioned in 50 ml serum borosilicate vials, poisoned with a saturated solution of HgCl_2_ (100 μL) and sealed with gas tight butyl stoppers until analysis by gas chromatography (GC)^54^. The CH_4_ partial pressure was measured in a 1 mL subsample of the headspace of 20 mL of N_2_ that was allowed to equilibrate about 12h after initial vigorous shaking. The CH_4_ concentrations in the Amazon were measured with a flame ionization detector (FID) with a Hewlett Packard 5890A GC calibrated with certified CH_4_:N_2_ mixtures (Air Liquide France) of 10 ppm and 200 ppm CH_4_. The CH_4_ concentrations in the Congo were measured with a SRI 8610C GC-FID calibrated with certified CH_4_:CO_2_:N_2_O:N_2_ mixtures (Air Liquide Belgium) of 1, 10, 30 and 509 ppm CH_4_. The overall precision of measurements was ±2% and ±4% for pCO_2_ and CH_4_, respectively. Additional data in the Amazon were digitalized with PlotDigitizer© from the plots of Richey *et al*.^55^. Data presented in Richey *et al*.^55^ were obtained by headspace technique and GC analysis, from April 1982 to August 1985 during 9 cruises upstream of Manaus, while data reported in the present study were acquired downstream of Manaus.

### Computation of tropical river CO_2_ efflux and error propagation

The air-water CO_2_ flux (*F*) was computed according to:


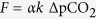


where *α* is the CO_2_ solubility coefficient, *k* is the gas transfer velocity and ΔpCO_2_ is the pCO_2_ air-water gradient, whereby a positive value corresponds by convention to an emission of CO_2_ from the water to the atmosphere.

**Figure 4:**
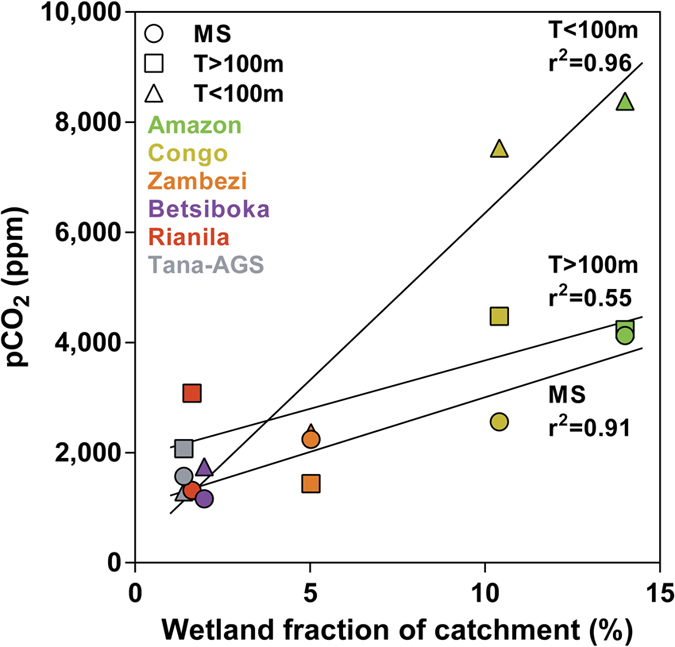
Median river-channel pCO_2_ in mainstem (MS), large tributaries (T > 100 m) and small tributaries (T < 100 m) as function of wetland coverage (fraction of the catchment) in the Amazon (n = 136), Congo (n = 280), Zambezi (n = 153), Betsiboka (n = 21), Rianila (n = 9), and Tana and Athi−Galana−Sabaki (Tana/AGS) (n = 442). Solid lines indicate linear regressions, and r^2^ are the corresponding coefficient of determination. Amazon and Congo data are from the present study, other data from Borges *et al*.^27^.

We used the geographical information system (GIS) of Raymond *et al*.^1^. The GIS provides *k* values, surface areas and width for streams and rivers globally, and the data are structured by stream order into COSCATs (coastal segmentation and its related catchment^56^). The *k* values themselves are derived from a parameterization as a function of slope and stream velocity^57^ included in the GIS. For each of the COSCAT units we derived wetland cover from another GIS, the global database of lakes, reservoirs and wetlands^58^. Based on the wetland coverage and the equations of the regressions in Fig. 4, we computed the water pCO_2_ in MS, T > 100m and T < 100m. Since river/stream surface areas in the GIS are structured by stream order it is not possible to distinguish the surface areas corresponding to MS and tributaries. So, the pCO_2_ of MS and T > 100m computed from the regressions for each COSCAT were averaged, and computations were further carried for T < 100m and for MS and T > 100m lumped together. The *F* values were then computed from the *k* values derived from the GIS for streams/rivers narrower and wider than 100 m, a constant water temperature of 25 °C to compute *α*^59^ and a constant atmospheric pCO_2_ of 390 ppm. The *F* areal values per COSCAT were scaled to the respective stream/river surface area and the data between 30°N and 30°S were summed to provide a total flux value for tropical areas.

An error analysis on the CO_2_ flux computation and upscaling was carried out by error propagation of the pCO_2_ computation, the *k* value estimates, and the estimate of surface areas of river channels to scale the areal fluxes, using a Monte Carlo simulation with 1000 iterations. The uncertainty on the pCO_2_ computation was derived from the errors on the slope and Y-intercept of the linear regressions in Fig. 4. The uncertainty on *k* values from the GIS was estimated to be ±10.0% based on the errors on slope and constant of the parameterization^57^. The river/stream surface areas in the GIS were estimated using two different hydraulic equations, that allow to estimate an uncertainty of ±31.0%.

### Statistical analysis

The statistical tests were done with GraphPad Prism® Version 6.05 for Windows.

### Original data-set

The timestamped and geo-referenced data-set of pCO_2_ and CH_4_ concentrations (Table 2) are available as a supplementary table.

## Additional Information

**How to cite this article**: Borges, A. V. *et al*. Divergent biophysical controls of aquatic CO_2_ and CH_4_ in the World’s two largest rivers. *Sci. Rep*. **5**, 15614; doi: 10.1038/srep15614 (2015).

## Supplementary Material

Supplementary Information

## Figures and Tables

**Table 1:** Main characteristics of the Amazon and Congo basins.

	Amazon	Congo
Catchment area (km^2^)^62^	6,025,735	3,705,222
Slope (°)^62^	1.4	0.6
Discharge (km^3^ yr^−1^)^63^	5,444	1,270
Specific discharge (L s^−1^ km^−2^)	29	11
Precipitation (mm)^64^	2,147	1,527
Air temperature (°C)^64^	24.6	23.7
River-stream surface area (km^2^)^1^	74,904	26,517
Wetland surface area (%)^11^,^58^	14	10
Above ground biomass (Mg km^−2^)^65^	909	748
Land cover^60^,^61^		
Dense Forest (%)	83	49
Mosaic Forest (%)	4	18
Woodland and shrubland (%)	4	27
Grassland (%)	5	3
Cropland/Bare soil (%)	4	2

**Table 2:** Dates, river stage, spatial coverage and number (*n*) of paired samples of pCO_2_ and CH_4_ collected in the Amazon and Congo rivers.

Dates	River stage	Longitude (°E)	Latitude (°N)	*n*
Amazon				
30/01/2007–09/02/2007	Rising water	−55.769; −51.239	−2.546; −0.116	28
11/05/2008–28/05/2008	High water	−60.920; −60.174	−3.397; −3.076	36
06/10/2008–13/10/2008	Low water	−60.291; −55.288	−3.410; −1.913	14
03/10/2009–20/10/2009	Low water	−60.824; −55.029	−3.467; −1.951	36
25/08/2010–12/09/2010	Falling water	−60.852; −54.988	−3.384; −1.947	22
Congo				
20/11/2012–08/12/2012	High water	24.170; 25.196	0.490; 0.795	32
17/09/2013–26/09/2013	Low water	24.169; 24.599	0.494; 0.775	6
03/12/2013–19/12/2013	High water	15.350; 25.187	−4.307; 2.206	75
13/03/2014–21/03/2014	High water	24.170; 24.604	0.493; 0.784	20
10/06/2014–30/06/2014	Falling water	15.357; 25.187	−4.306; 2.217	89
16/04/2015–06/05/2015	Falling water	15.392; 20.578	−4.394; 2.666	58
